# Women With Moderate or Severe Aortic Stenosis by Aortic Valve Area Are Disproportionately Classified With Normal‐Flow Low‐Gradient Aortic Stenosis and Assigned Lower Severity Grades Than Men

**DOI:** 10.1111/echo.70270

**Published:** 2025-08-19

**Authors:** Christopher Sidwell, Anna Curtis, Theodore Kolias, Andrew Harris, Megan Joseph, Troy LaBounty

**Affiliations:** ^1^ Department of Internal Medicine University of Michigan Ann Arbor USA; ^2^ Department of Cardiovascular Medicine Cleveland Clinic Cleveland USA

**Keywords:** aortic valve, aortic stenosis, gender, sex

## Abstract

**Purpose:**

Patients with aortic stenosis (AS) may have discordant severity grades between AS by aortic valve area (AS_AVA_) and AS using hemodynamic‐based guidelines (AS_GL_). Individuals with normal left ventricular function and normal‐flow low‐gradient (NF‐LG) AS that is moderate or severe by AVA are downgraded in severity by current guidelines. We evaluated the prevalence and risk of NF‐LG AS in patients with moderate or severe AS_AVA_.

**Methods:**

We compared the prevalence and mortality of NF‐LG AS and low‐flow low‐gradient (LF‐LG) AS compared to normal‐flow high‐gradient (NF‐HG) AS in patients with moderate and severe AS_AVA_ and normal left ventricular function.

**Results:**

Among 5360 patients, 2974 had moderate AS_AVA_ and 2386 had severe AS_AVA_ (mean age 73.3 ± 12.8 years; 49.3% female; 1637 deaths over median of 4.2 years). In moderate AS_AVA_, women were more frequently classified as NF‐LG AS, with AS severity downgraded to mild using guidelines (52.0% women vs. 29.1% men, *p* < 0.001). In severe AS_AVA_, women were also more likely to have NF‐LG AS and be classified as mild or moderate AS using guidelines (23.9% women vs. 14.4% men, *p* < 0.001). Patients with NF‐LG moderate or severe AS_AVA_ had comparable mortality to NF‐HG AS (*p* > 0.05 for each).

**Conclusion:**

Women with moderate or severe AS_AVA_ have markedly higher rates of NF‐LG AS than men and are more likely to be assigned a lower AS severity grade based on current guidelines, despite mortality similar to NF‐HG AS. Discordance in AS severity between AS_AVA_ and AS_GL_ is common in both genders, although more common in women.

## Graphical abstract

We observed that there are gender discrepancies between aortic stenosis phenotypes, with females comprising a disproportionate number of NF‐LG cases; we also observed that women are more likely than men to have discordant aortic stenosis severity between AS by AVA vs AS by guidelines, despite comparable mortality.

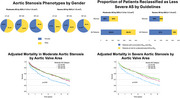

**Abbreviations**
AS: aortic stenosis
AS_AVA_
: aortic stenosis by aortic valve area
AS_GL_
: aortic stenosis by guidelines
LF‐LG: low flow, low gradient
NF‐HG: normal flow, high gradient
NF‐LG: normal flow, low gradient

## 1 Introduction

Aortic stenosis (AS) is a common valve disease with a high rate of mortality when not treated promptly, and undertreatment remains common [1]. Managing patients with aortic stenosis (AS) is highly dependent on the severity of the disease, and the diagnostic criteria for AS severity have changed over time. The most current guidelines (2020 American Heart Association/American College of Cardiology) [2] utilize aortic valve area (AVA) as a diagnostic criterion for only severe AS, while previous guidelines from other societies (2017 European Association of Cardiovascular Imaging/American Society of Echocardiography) additionally incorporated AVA to define mild and moderate AS [3]. These changes were made in part to address concerns that the calculated AVA is susceptible to error. The most current guidelines require patients to meet certain criteria in addition to AVA to be defined with severe AS, such as requiring a low stroke volume index (SVI) in patients with normal LV systolic function and an AVA ≤1.0 cm^2^ if the mean pressure gradient is <40 mmHg and the peak velocity is <4.0 m/s; otherwise, they would be reclassified as mild or moderate AS. Similarly, patients previously diagnosed with moderate AS based on an AVA <1.5 cm^2^ may now be defined as having mild AS if the mean pressure gradient is <20 mmHg and the peak velocity is <3.0 m/s.

We characterized patients with normal LV systolic function and moderate or severe AS based on AVA (AS_AVA_) with normal‐flow low‐gradient (NF‐LG), low‐flow low‐gradient (LF‐LG), or normal‐flow high‐gradient (NF‐HG) AS. Patients with moderate NF‐LG AS_AVA_ would be reclassified as mild AS by current guidelines (AS_GL_), while those with severe NF‐LG AS_AVA_ would be reclassified as mild or moderate AS_GL_. While LF‐LG moderate AS is not described in current guidelines, it is common and is associated with increased mortality [4], and was included in these analyses. We examined the frequency with which patients would be reclassified from severe to mild or moderate AS or from moderate to mild AS between AS_AVA_ and AS_GL_, and specifically evaluated whether there was a gender disparity in how often this reclassification occurred. Our hypothesis was that women would be more likely than men to be reclassified with a lower grade of AS severity using current guidelines.

## 2 Methods

We evaluated consecutive patients undergoing echocardiography between October 2012 and October 2022 at a single academic medical center. Inclusion criteria included adult patients with a complete transthoracic echocardiogram, at least moderate anatomic AS (AVA ≤1.5 cm^2^), and normal LV systolic function (ejection fraction ≥50%). Patients with prior surgical or transcatheter aortic valve replacement were excluded. From a total cohort of 150,040 patients, 5360 subjects met the inclusion criteria and none of the exclusion criteria. For patients with multiple echocardiogram studies, the initial study was used.

To examine the effect of reclassification between prior and current guidelines, we characterized patients as having moderate (AVA >1.0 to 1.5 cm^2^) or severe (≤1.0 cm^2^) AS_AVA_. Patients were then stratified according to current guidelines as having NF‐LG AS, LF‐LG AS, or NF‐HG AS. Low‐flow was defined as SVI <35 mg/m^2^. High‐gradient AS was defined as a mean aortic valve pressure gradient of 20–39 mmHg or a peak aortic valve velocity of 3.0–3.9 m/s for moderate AS, or a mean pressure gradient of ≥40 mmHg or a peak velocity of ≥4.0 m/s for severe AS.

Echocardiograms were performed at a tertiary care academic medical center, one of 6 affiliated satellite clinics located in 5 cities, and an affiliated private practice primary care hospital in a sixth city; sites were located within three adjacent counties. All studies were interpreted by a core group of experienced cardiologists with Level III training in echocardiography in a central accredited laboratory with rigorous quality control using a standard protocol. Echocardiography performance followed recommended standardized guidelines and included a comprehensive and standardized study of all cardiac structures, including a thorough evaluation of aortic valve gradients from all possible windows and Doppler interrogation of the aortic valve with a nonimaging probe when possible [5]. Echocardiograms were performed using Philips EPIQ 7 and iE33 systems (Philips Healthcare, Andover, MA, USA), or Acuson Sequoia 512 systems (Siemens, Malvern, PA, USA), and archived in standard DICOM format. Images were reviewed using contemporary versions of Synapse Cardiovascular Client (Fujifilm Medical Systems, Valhalla, NY, USA). Echocardiography measurements were performed in accordance with guidelines [5, 6].

Clinical data were extracted from the electronic medical record. All‐cause mortality was assessed within the electronic medical record based on its query of state and federal death records.

Chi‐square tests were used to compare categorical variables. Continuous variables were evaluated using one‐way ANOVA for variables with normal distributions, or an Independent Samples Kruskal–Wallis test for variables without a normal distribution. Cox regression analyses were used to compare the relationship between AS category and mortality. Multivariable analyses adjusted for age, gender, diabetes, hypertension, hyperlipidemia, prior myocardial infarction, coronary artery disease, current tobacco smoking, or history of stroke/transient ischemic attack. *p* values <0.05 were considered significant. Statistical analyses were performed using IBM SPSS version 30 for Mac (IBM Corporation; Armonk, NY, USA).

## 3 Results

Mean age was 73.3 ± 12.8 years and 49.3% (2642/5360) were female. There were 1637 deaths and with a median follow‐up of 4.2 years. From the cohort of 5360 patients, 55.5% (*n* = 2974) had moderate AS_AVA,_ and 44.5% (*n* = 2386) had severe AS_AVA_. Patient characteristics are provided in Tables 1 and 2.

**TABLE 1:** Baseline characteristics for patient with moderate aortic stenosis by aortic valve area.

	Low‐flow, low‐gradient *n*=687	Normal‐flow, low‐gradient *n*=1203	Normal‐flow, high‐gradient *n*=1084	*p*
**Demographics**				
Age (years)	72.7 ± 12.5	74.9 ± 12.0	69.9 ± 13.4	<0.001
Female gender (%)	42.8	63.7	38.1	<0.001
Body mass index (kg/m^2^)	31.4 ± 7.7	27.8 ± 5.9	30.7 ± 6.9	<0.001
Body surface area (m^2^)	2.0 ± 0.2	1.8 ± 0.2	2.0 ± 0.3	<0.001
**Medical history**				
Diabetes (%)	42.8	36.4	41.4	0.01
Hypertension (%)	82.3	84.5	75.7	0.14
Hyperlipidemia (%)	73.5	74.2	72.7	0.71
Myocardial infarction (%)	24.9	22.7	20.2	0.06
Coronary artery disease	59.1	53.3	60.4	0.001
Tobacco smoking (%)	27.2	24.7	30.3	0.01
Stroke or TIA (%)	20.8	20.2	17.6	0.17
**Echo variables, median (interquartile range)**				
Ejection fraction (%)	63 (60–65)	65 (60–65)	65 (60–70)	<0.001
Stroke volume index (mL/m^2^)	27 (31–33)	38 (42–46)	39 (45–51)	<0.001
AV peak velocity (m/s)	2.3 (2.2–2.5)	2.5 (2.3–2.7)	3.3 (3.1–3.6)	<0.001
AV mean gradient (mmHg)	11 (10–14)	14 (11–16)	24 (21–28)	<0.001
AV area (cm^2^)	1.2 (1.1–1.4)	1.3 (1.2–1.4)	1.2 (1.1–1.3)	<0.001

*Note*: Variables provided as percentage, mean ± standard deviation, or median (interquartile range).

Abbreviations: AV, aortic valve; TIA, transient ischemic attack.

**TABLE 2:** Baseline Characteristics for Patient with Severe Aortic Stenosis by Aortic Valve Area.

	Low‐flow, low‐gradient *n*=602	Normal‐flow, low‐gradient *n*=455	Normal‐flow, high‐gradient *n*=1329	*p*
**Demographics**				
Age (years)	76.8 ± 12.1	77.2 ± 11.6	72.1 ± 13.1	<0.001
Female gender (%)	51.0	61.5	44.0	<0.001
Body mass index (kg/m^2^)	29.1 ± 6.8	26.9 ± 6.0	30.5 ± 7.6	<0.001
Body surface area (m^2^)	1.9 ± 0.3	1.8 ± 0.3	1.9 ± 0.3	<0.001
**Medical history**				
Diabetes (%)	40.0	34.7	37.1	0.20
Hypertension (%)	85.1	84.8	84.0	0.82
Hyperlipidemia (%)	76.6	75.4	72.1	0.08
Myocardial infarction (%)	23.9	20.1	18.1	0.01
Coronary artery disease (%)	68.6	64.0	65.7	0.26
Tobacco smoking (%)	29.9	25.1	31.4	0.04
Stroke or TIA (%)	23.1	18.2	18.4	0.04
**Echo variables, median (interquartile range)**				
Ejection fraction (%)	61 (60–65)	65 (60–68)	65 (60–70)	<0.001
Stroke volume index (mL/m^2^)	30 (25–33)	40 (38–45)	41 (34–50)	<0.001
AV peak velocity (m/s)	3.2 (2.7–3.6)	3.5 (3.2–3.8)	4.4 (4.2–4.8)	<0.001
AV mean gradient (mmHg)	23 (16–29)	28 (23–32)	45 (40–54)	<0.001
AV area (cm^2^)	0.79 (0.69–0.89)	0.89 (0.82–0.95)	0.76 (0.61–0.92)	<0.001

Variables provided as percentage, mean ± standard deviation, or median (interquartile range).

Abbreviations: AV, aortic valve; TIA, transient ischemic attack.

Overall, moderate AS_AVA_ would be reclassified as mild AS using current guidelines in 40.4% (1203/2974) of patients; severe AS_AVA_ would be reclassified as mild or moderate using current guidelines in 19.0% (455/2386) of patients.

**FIGURE 1:**
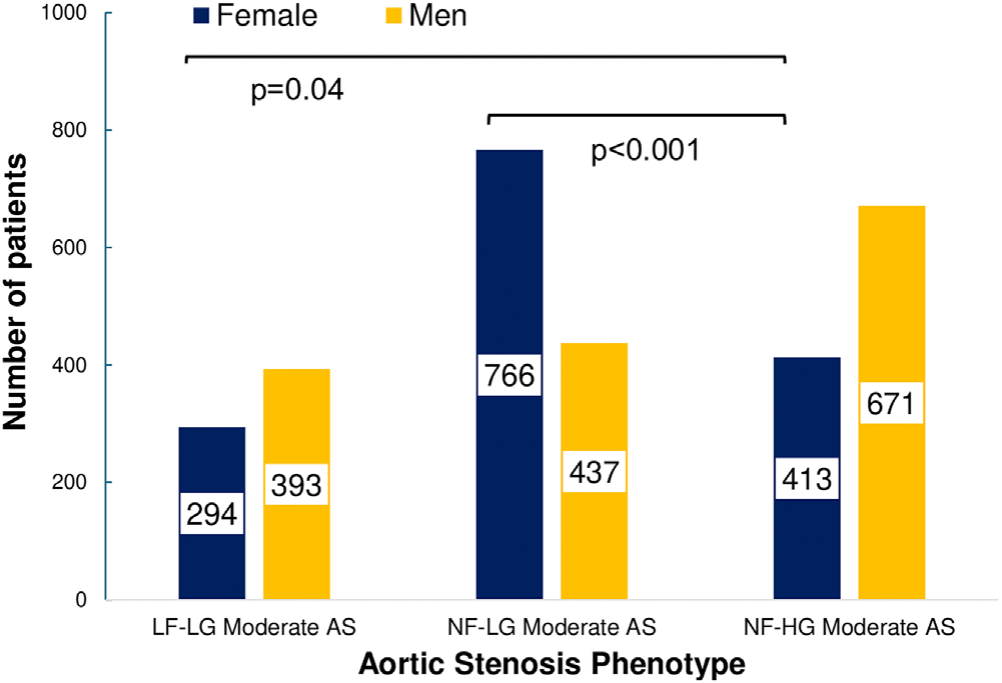
Categories of moderate AS by aortic valve area. AS, aortic stenosis; LF‐LG, low‐flow, low‐gradient; NF‐HG, normal‐flow, high‐gradient; NF‐LG, normal‐flow, low‐gradient.

**FIGURE 2:**
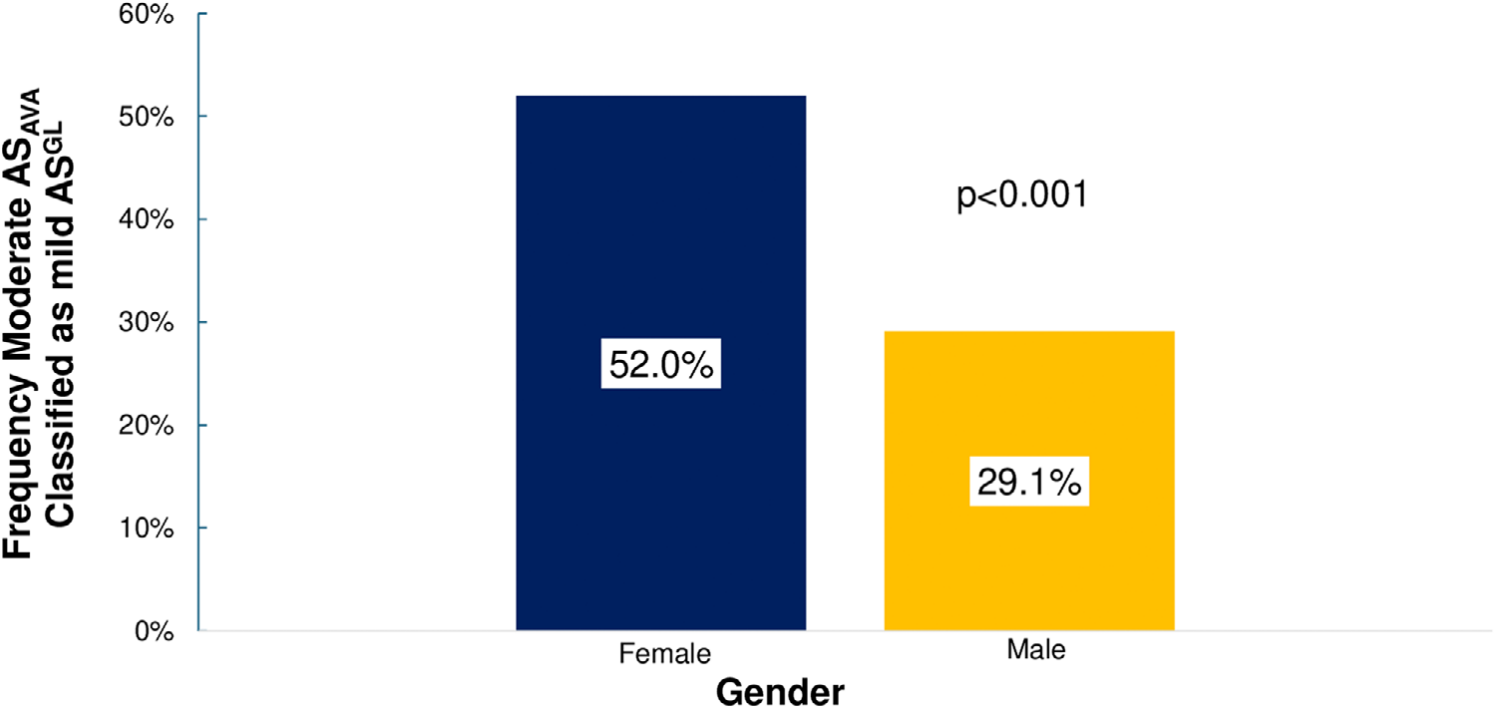
Gender‐specific frequency that moderate AS by aortic valve area is classified as mild AS using current guidelines. AVA, aortic valve area; AS, aortic stenosis.

In patients with moderate AS_AVA_, 49.5% (*n* = 1473) were female. Within AS categories for patients with moderate AS_AVA_ (Figure 1), women were more likely than men to meet criteria for NF‐LG AS (63.7% vs. 36.3%, *p* < 0.001 vs. other categories). Women represented 42.8% of LF‐LG patients (vs. 57.2% for men), and 38.0% of NF‐HG patients (vs. 62.0% for men), with significant differences in gender distributions compared to NF‐HG moderate AS_AVA_, as demonstrated in the figure. Women with moderate AS_AVA_ were more likely than men to be downgraded to mild AS using current guidelines (Figure 2).

**FIGURE 3:**
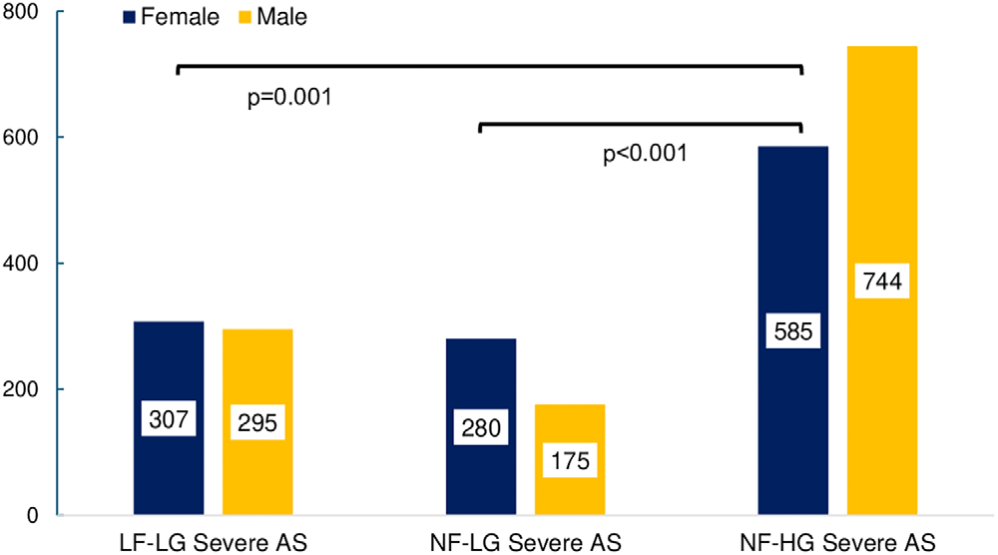
Categories of severe AS by aortic valve area. AS, aortic stenosis; LF‐LG, low‐flow, low‐gradient; NF‐LG, normal‐flow, low‐gradient; NF‐HG, normal‐flow, high‐gradient.

**FIGURE 4:**
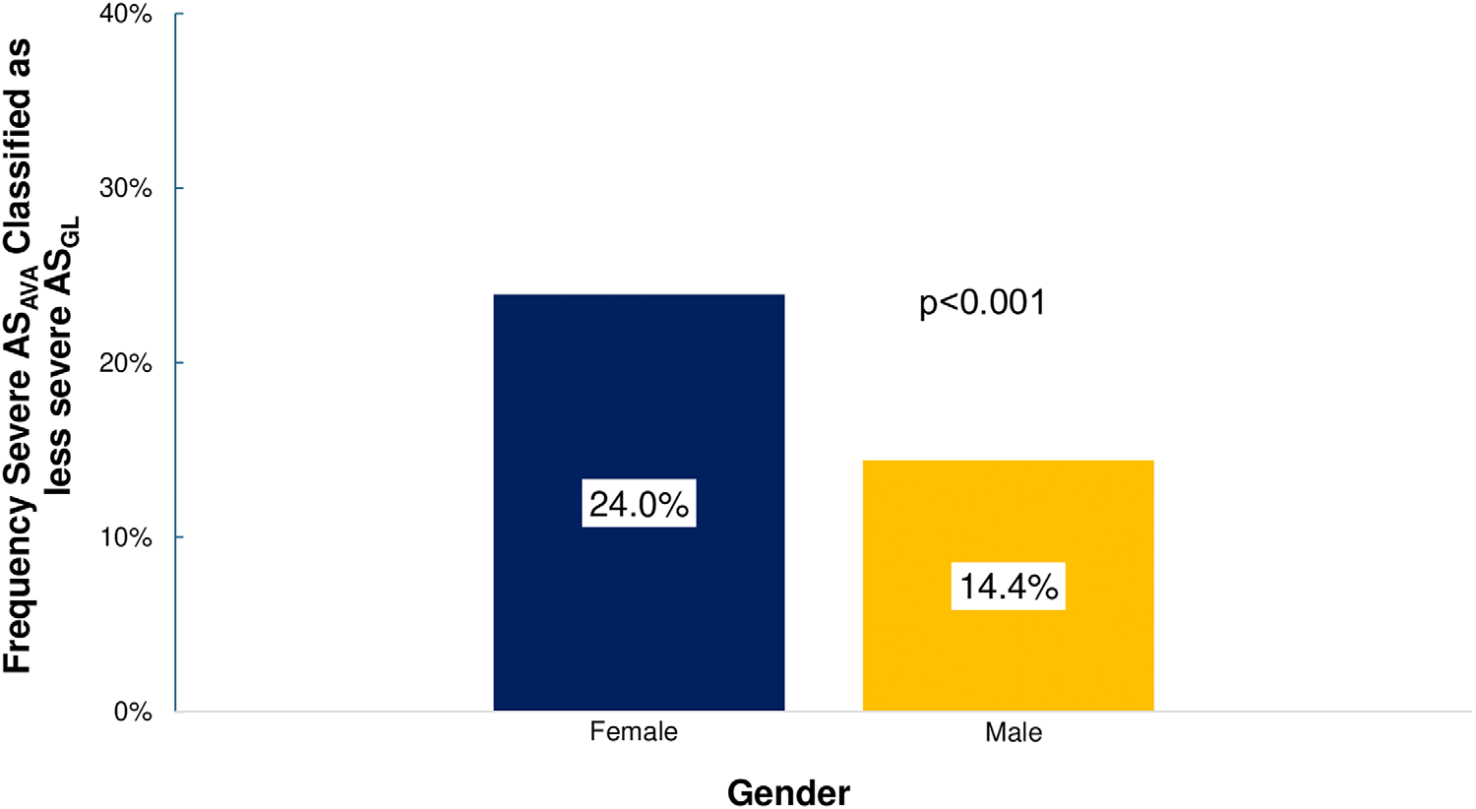
Gender‐specific frequency that severe AS by aortic valve area is classified as mild or moderate AS using current guidelines. AVA, aortic valve area; AS, aortic stenosis.

In patients with severe AS_AVA_, 49.1% (*n* = 1172) were female. Within categories of severe AS_AVA_ (Figure 3), women were more likely than men to meet criteria for NF‐LG AS (61.6% vs. 38.4%, *p *< 0.001 vs. other categories). Women represented 51.0% of LF‐LG patients (vs. 49.0% for men), and 44.0% of NF‐HG patients (vs. 56.0% for men), with significant differences in gender distributions compared to NF‐HG severe AS, as demonstrated in the figure. Women with severe AS_AVA_ were more likely to be downgraded to mild or moderate AS compared to men using current guidelines (Figure 4).

**FIGURE 5:**
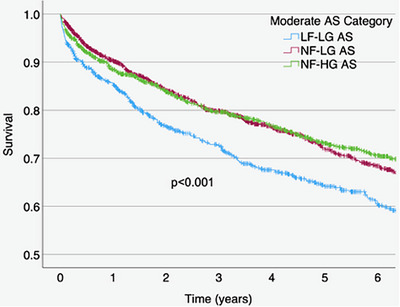
Kaplan‐Meier curve for mortality in patients classified as LF‐LG, NF‐LG, and NF‐HG moderate aortic stenosis by aortic valve area. Log‐rank *p* < 0.001. LF‐LG, low‐flow, low‐gradient; NF‐HG, normal‐flow, high‐gradient; NF‐LG, normal‐flow, low‐gradient.

There were differences in mortality between groups with moderate AS_AVA_ (*p* < 0.001), with the highest mortality in those with LF‐LG AS_AVA_ (Figure 5). Adjusted mortality (Table 3) remained higher for those with moderate LF‐LG AS compared to NF‐HG AS, while individuals with NF‐LG AS had similar mortality. Among women with moderate AS_AVA_, there were no significant differences in mortality between groups, while men with LF‐LG had higher mortality compared to NF‐HG AS.

**TABLE 3:** Adjusted mortality in moderate aortic stenosis by aortic valve area.

	Hazard ratio	95% CI	*p* value
**Overall**			
NF‐HG AS	—	—	—
LF‐LG AS	1.27	1.06–1.52	0.008
NF‐LG AS	0.92	0.78–1.09	0.30
**Women**			
NF‐HG AS	—	—	—
LF‐LG AS	1.29	0.99–1.69	0.06
NF‐LG AS	0.82	0.65–1.03	0.09
**Men**			
NF‐HG AS	—	—	—
LF‐LG AS	1.27	1.01–1.58	0.04
NF‐LG AS	1.03	0.81–1.23	0.81

*Note*: NF‐HG severe AS used as reference.

Abbreviations: AS, aortic stenosis; LFLG, low‐flow, low‐gradient; NF‐HG, normal‐flow high‐gradient; NF‐LG, normal‐flow low‐gradient.

**FIGURE 6:**
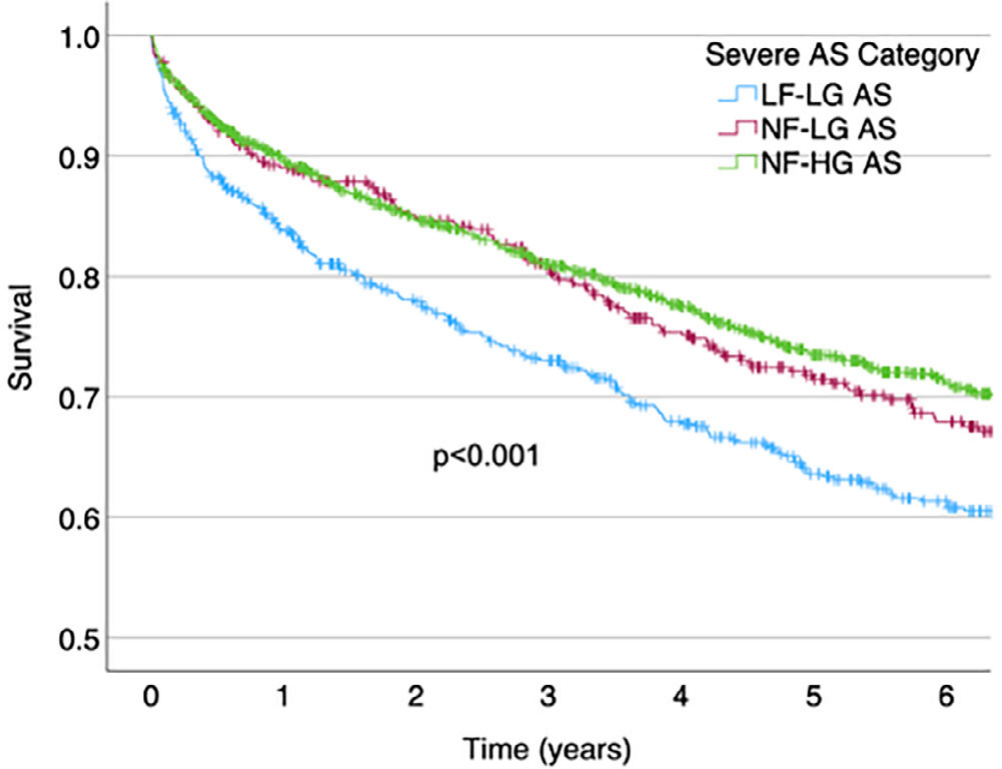
Kaplan‐Meier curve for mortality in patients classified as LF‐LG, NF‐LG, and NF‐HG severe aortic stenosis by aortic valve area. Log‐rank *p* < 0.001. LF‐LG, low‐flow, low‐gradient; NF‐HG, normal‐flow, high‐gradient; NF‐LG, normal‐flow, low‐gradient.

Mortality differences were also observed between groups with severe AS_AVA_ (*p* < 0.001), with the highest mortality in patients with LF‐LG AS_AVA_ (Figure 6). Adjusted mortality (Table 4) was also higher in patients with severe LF‐LG AS_AVA_ compared to NF‐HG AS, while patients with NF‐LS AS had similar mortality. Men with severe LF‐LG AS_AVA_ had increased mortality compared to those with NF‐HG AS, with no other significant differences observed between genders.

**TABLE 4:** Adjusted mortality in severe aortic stenosis by aortic valve area.

	Hazard ratio	95% CI	*p* value
**Overall**			
NF‐HG AS	—	—	—
LF‐LG AS	1.28	1.08–1.53	0.006
NF‐LG AS	0.95	0.77–1.17	0.60
**Women**			
NF‐HG AS	—	—	—
LF‐LG AS	1.26	0.99–1.59	0.05
NF‐LG AS	0.89	0.67–1.18	0.42
**Men**			
NF‐HG AS	—	—	—
LF‐LG AS	1.30	1.03—‐1.64	0.03
NF‐LG AS	1.01	0.73–1.38	0.99

*Note*: NF‐HG severe AS used as reference.

Abbreviations: AS, aortic stenosis; LFLG, low‐flow, low‐gradient; NF‐HG, normal‐flow high‐gradient; NF‐LG, normal‐flow low‐gradient.

## 4 Discussion

The application of current guidelines frequently downgrades AS severity compared to AS_AVA_, particularly in women. We observe that over half of women with moderate AS_AVA_ (52%) are reclassified as mild AS using current guidelines, if an AVA of <1.5 cm^2^ is no longer included as part of the definition of moderate AS. Further, a significant proportion of women with severe anatomic AS (24%) would be reclassified as mild or moderate AS despite an AVA ≤1.0 cm^2^, as they do not meet the cutoff to define LF‐LG severe AS (SVI <35 mL/m^2^). Men are also frequently reclassified to lower AS severity grades (29% and 14% of men with moderate or severe AS_AVA_, respectively), although at a lower frequency than women.

The greater emphasis on valve hemodynamics in current guidelines acknowledges the limitations of using AVA in grading severity, as this calculation is susceptible to several errors. Geometric assumptions and measurement errors in the left ventricular outflow tract diameter can result in significant errors. Further, errors in performing or measuring the left ventricular outflow tract or aortic valve spectral Doppler measurements can under‐ or over‐estimate the AVA. Nevertheless, when performed carefully, the AVA generally correlates well with invasive hemodynamics [7], and a smaller AVA is associated with increased mortality [8, 9, 10, 11, 12]. Consistent with this, we observed that patients with moderate and severe NF‐LG AS_AVA_—who would be reclassified as mild or moderate AS using current guidelines—had comparable mortality to moderate and severe NF‐HG AS patients, suggesting that these patients are not a low‐risk cohort and merit further investigation.

Patients with NF‐LG AS were slightly smaller in body size than the other cohorts in both the moderate and severe AS_AVA_ patients (based on body mass index and body surface area). In current guidelines, LF‐LG AS requires an SVI <35 mL/m^2^ in patients with normal left ventricular function. Smaller patients will have a larger SVI given their smaller body surface area, and some patients with SVI close to 35 could have their classification shifted based on their body size, making it harder for smaller patients to achieve a SVI <35 mL/m^2^. Further, larger patients may have a smaller SVI, making it easier for larger patients to achieve this cutoff. This could explain part of the findings in this manuscript, and future studies evaluating the optimal SVI cutpoint to use in patients with low or high body weights may be warranted.

There are many potential differences in the phenotypes of women with AS. Women with AS have been reported to have slower progression in disease severity and less valve calcification, but have higher left ventricular ejection fraction and increased rates of concentric left ventricular hypertrophy compared to men, while men are more likely to have left ventricular dysfunction and dilatation [13, 14]. Women are diagnosed at later ages than men despite having more symptoms but are more likely to be undertreated [15]. Women with AS are more likely to have diastolic dysfunction, myocardial fibrosis, hypertension, reduced arterial compliance, and reduced left ventricular compliance, which reflect significant gender‐specific differences in the myocardial and vascular response to AS [16]. Whether our observed findings are related to these many differences or due to other causes remains unclear. Our findings suggest that AS severity in women may be underestimated more frequently than in men and could result in delayed or missed diagnoses in women with AS. Reconsidering the role of AVA in defining AS severity may be warranted and may merit further study. While we recognize the potential for errors in the AVA, it remains a useful calculation and could be considered for a secondary role in future guidelines, especially in cases with concern for underestimation of AS severity.

When there is discordance between AS severity using current guidelines and the AS_AVA_, the best approach is not clear. For example, a woman with dyspnea on exertion, normal left ventricular systolic function, AVA of 0.8 cm^2^, aortic valve mean gradient of 18 mmHg, peak velocity of 2.6 m/s, and a stroke volume index of 42 mL/m^2^ would be characterized as mild AS by current guidelines, despite potentially severe AS_AVA_. While guidelines emphasize the importance of evaluating each echo individually and using guidelines as a “guide” only, using the current criteria could result in significant underestimation of AS severity. Based on our findings, significant underestimation of AS severity is potentially common, particularly in women.

Underestimating AS severity may have significant adverse consequences. A symptomatic patient with an echocardiogram reporting only mild AS may undergo a workup for alternative causes of the symptoms, and could have delays to diagnosis and treatment if the AS was, in fact, significant. Further, surveillance imaging is often performed in patients with valvular heart disease, and underestimation of AS severity could result in inadequate surveillance imaging for progression and delays in identifying hemodynamically significant AS. Given the high mortality burden of untreated or undertreated AS, delays to diagnosis and treatment may be associated with a significant risk of morbidity and mortality [1]. Future studies evaluating the benefits of surgical and transcatheter valve replacement in these specific cohorts in both genders may help identify patients who may most benefit from intervention and inform future guidelines [17].

There are several limitations of this study. The patient population represented a limited geographic area and a single academic center, which may limit application to other populations. Patients were included after referral for a clinically indicated echocardiogram, which may introduce selection bias, and studies were not blindly interpreted, which can introduce further bias. Further, measurements are susceptible to error and inter‐reader variability, which can impact the calculated AVA. As a result, some patients with AS defined by AVA may be incorrectly classified.

Patients with moderate or severe AS_AVA_ and normal left ventricular function are often reclassified as mild or moderate AS using current guidelines, with women disproportionately reclassified compared to men. Further study may be warranted to better understand the role of AS_AVA_ in defining AS severity, and future guidelines may benefit from assessing the gender‐specific impact of changes in how we define valve severity.

## Conflicts of Interest

The authors declare no conflicts of interest

## Ethics Statement

This study was approved by the University of Michigan Institutional Review Board.

## Data Availability

De‐identified data is available upon reasonable request.
